# Cultural representation, soft power, and tourism futures in Vision 2030: Saudi Arabia's path toward the 2034 FIFA World Cup

**DOI:** 10.3389/fsoc.2026.1761036

**Published:** 2026-05-19

**Authors:** Shuruq Alsharif

**Affiliations:** Sociology and Social Work Department, Imam Abdulrahman Bin Faisal University, Dammam, Saudi Arabia

**Keywords:** discourse analysis, FIFA World Cup 2034, gender policy, gender representation, rural-urban divide, social transformation, symbolic empowerment, tourism sector

## Abstract

This article provides one of the first systematic sociological analyses of Saudi women's representation and positioning within tourism and sports narratives linked to Vision 2030 and Saudi Arabia's preparations for hosting the FIFA World Cup 2034. Addressing a notable gap in scholarship, the study examines the relationship between gendered media discourse, public visibility, and state-led modernization. Grounded in cultural representation theory, feminist reconceptualizations of the public sphere, and gender performativity, the study employs critical discourse analysis to examine a curated corpus of national tourism advertisements, sports promotional campaigns, government communications, and institutional reports published between 2022 and 2025. The findings reveal a strategic transformation in the representation of Saudi women as public actors, entrepreneurs, athletes, and cultural ambassadors operating within tourism and international engagement spaces. These representations combine symbolic references to Saudi heritage, including the abaya, desert landscapes, and equestrian culture, with narratives emphasizing modernization, mobility, and global openness. However, the analysis also demonstrates that these transformations remain uneven, with women's visibility and participation appearing more pronounced in metropolitan and globally oriented spaces than in rural regions, where local cultural norms continue to shape the pace and form of social change. The study further suggests that while symbolic inclusion and public visibility have expanded considerably, deeper forms of structural empowerment remain dependent on institutional mechanisms capable of supporting leadership opportunities, public-private collaboration, and regionally responsive capacity-building strategies. By situating Saudi women at the intersection of gender politics, tourism development, nation branding, and global sports diplomacy, the article contributes to broader debates on symbolic power, visibility, and social transformation in the contemporary Middle East while offering insight into how the 2034 FIFA World Cup may further reshape gendered participation in Saudi Arabia's evolving global future.

## 1 Introduction

Amid the social and economic transformations driven by Saudi Arabia's Vision 2030, Saudi women's expanding participation in sectors historically dominated by men has become one of the most visible indicators of change. Labor-market data show that women's labor force participation has risen sharply since 2016, exceeding the initial Vision 2030 target of 30% and reaching the mid-30% range in recent years (U.S.-Saudi Business Council, 2023; Vision 2030, 2020–2024). Within this broader shift, tourism and hospitality have emerged as emblematic arenas of gendered transformation. This pattern is further supported by recent empirical research demonstrating that gender significantly shapes tourism motivations in Saudi Arabia, with women showing stronger engagement in culturally embedded and family-oriented tourism practices, while men tend to prioritize autonomy and social exploration (Alkohaiz et al., 2025). Official figures and policy briefings indicate that women now constitute a substantial share of the tourism workforce; the Ministry of Tourism has reported that women represent nearly half of all employees in the sector, following a rapid increase in hiring since 2018 (Ministry of Tourism, Saudi Arabia, 2024a; Saudi Press Agency, 2024). These developments align with national diversification strategies that frame women's economic participation as a core pillar of Saudi Arabia's post-oil transition.

Tourism is not only an economic growth engine but also a key instrument in shaping a national image. Residents' emotional responses to tourism development further indicate that tourism expansion is socially negotiated and may generate both support and ambivalence within local communities (Algassim et al., 2023). Tourism motivations in Saudi Arabia are shaped by both cultural norms and evolving lifestyle expectations, reflecting the intersection of tradition and modernization processes (Mansour and Mumuni, 2019). Tourism is closely linked to modernity, serving as a social space where identities are reconfigured, and individuals negotiate between tradition and contemporary social expectations (Wang, 2000). Tourist destinations are not perceived objectively but are constructed through appraisive images shaped by media representations and prior cognitive frameworks (Walmsley and Jenkins, 1993). The National Tourism Strategy explicitly casts tourism as a vehicle for re-positioning Saudi Arabia as an open culturally rich destination while maintaining its religious and social distinctiveness (Ministry of Tourism, Saudi Arabia, 2019; Vision 2030, 2020–2024). In this context, Saudi women are increasingly placed at the center of branding narratives: as front-line staff in hospitality; as heritage interpreters at sites such as AlUla; and as visible figures in promotional campaigns that present them as professional, confident, and rooted in local culture. These state-supported narratives link gender inclusion with “modern authenticity,” signaling that women's participation is not an add-on to development but part of the symbolic core of how the nation is re-imagined for global audiences.

Parallel shifts are taking place in sports, especially football. Since the launch of the Saudi Women's Premier League in 2022, women's participation in organized football has expanded, with the women's game attracting both institutional backing and increased fan engagement (Arab News, 2025a). Recent studies indicate that a clear majority of women sports fans in the Kingdom follow football, underscoring the sport's cultural reach among women as spectators as well as participants (Arab News, 2025b). Football thus functions as a highly visible arena where female presence is not only tolerated but promoted, intersecting with broader state efforts to use sport as a tool of cultural diplomacy and global positioning. This momentum is substantiated by NEOM's comprehensive report “Pioneering Change: Women's Football in Saudi Arabia,” which documents remarkable achievements from 2018–2024 (NEOM Sport, and Asian Football Confederation, 2025). Key milestones include the formation of the national women's team in 2021, a 300% growth in participating teams across five domestic competitions, and the involvement of 77,000 schoolgirls in league play-the highest recorded figure. The sport has also seen 35 internationally certified female referees and 1,000 qualified coaches, alongside a 773% surge in girls aged 6–17 attending regional training centers. These developments, supported by Vision 200′s infrastructure investments and partnerships, like those with the Asian Football Confederation, signal Saudi Arabia's ambition to become a global leader in women's football ahead of hosting the 2034 FIFA World Cup (NEOM Sport, and Asian Football Confederation, 2025).

These economic and symbolic shifts have generated a substantial body of research on women's empowerment in Saudi Arabia under Vision 2030. Existing studies have examined women's employment and career trajectories in tourism and hospitality (Sobaih and Abu Elnasr, 2024), their emerging leadership in sports institutions (Alhazza, 2024), and the broader expansion of women's participation in sports and public life. They have also addressed the role of legal and digital reforms, such as the relaxation of male guardianship rules and the expansion of mobile government platforms, in widening women's capabilities to travel, work, and access public services (Alotaibi et al., 2025; Alshahrani and Ally, 2018). Qualitative work using Nobel Laureate Amartya Sen's capability approach shows that initiatives like the Absher platform and Yesser program enable many women to secure driving licenses, passports, and educational opportunities independently, while at the same time revealing how family norms, local religious interpretations, and digital literacy continue to mediate the practical extent of autonomy (Alotaibi et al., 2025). This literature points to a pattern by which women's visibility and numerical participation advance faster than deeper shifts in power relations within organizations, families, and public space. Community-based tourism research further demonstrates that women's early inclusion and direct participation in tourism activities can facilitate the renegotiation of gender norms and contribute to more inclusive development trajectories (Nikjoo et al., 2025). At the level of tourism behavior, similar gendered distinctions have been empirically observed, where women's participation is more closely tied to collective and culturally grounded practices, while men's motivations emphasize independence and experiential exploration (Alkohaiz et al., 2025).

At the same time, tourism in Saudi Arabia is increasingly entangled with mega-event ambitions and global sports diplomacy. The awarding of the 2034 FIFA World Cup to Saudi Arabia marks a critical moment in which sport, tourism, and national branding converge (Fédération Internationale de Football Association, 2023). Mega-events on this scale are not purely sporting competitions; they are designed and governed as platforms for projecting desired national images, attracting investment, and reconfiguring urban and social spaces. In the Saudi case, official campaigns already link women's presence in tourism and sports to narratives of openness, youthfulness, and reform (Ministry of Tourism, Saudi Arabia, 2024a). However, current scholarship has not yet systematically addressed how such mega-event futures might reshape Saudi women's roles in tourism labor markets, public space, and international representation, or whether these roles will be primarily symbolic or substantively empowering.

This gap is particularly striking given parallel debates in Gulf and broader Middle Eastern scholarship. Research on Saudi women's participation in the public domain has shown how women navigate and renegotiate boundaries of space, visibility, and respectability in ways that are locally grounded rather than merely derivative of Western feminist models (Le Renard, 2008, 2014; van Geel, 2016). Studies of women's employment and education highlight the tension between formal openings created by state reform and the everyday negotiations within families and communities that shape what is actually possible for women to do (Alhawsawi and Jawhar, 2023; Hamdan, 2005). Analyses of state-led “empowerment” agendas in development policy warn that women's inclusion is often mobilized instrumentally to signal modernity without necessarily redistributing power or addressing structural inequality (Cornwall and Rivas, 2015; World Bank, 2013). Yet there is still no focused examination of how these dynamics play out at the intersection of gender, tourism development, and mega-event politics in Saudi Arabia. While existing studies have examined gendered tourism motivations in isolation, including recent large-scale survey-based analyses (Alkohaiz et al., 2025), the interaction between these behavioral patterns and state-driven representational strategies in mega-event contexts remains underexplored.

The present study addresses this gap through a prospective, critically oriented analysis of Saudi women's positioning within tourism and sports narratives in the run-up to the 2034 World Cup, and how these positions relate to broader questions of social and cultural sustainability under Vision 2030. Rather than treating the increased visibility of women as self-evident progress, the article interrogates how women are represented, where they are located in tourism and sports ecologies, and what kinds of agency are made imaginable or foreclosed. It brings together sectoral data, state strategies, and media campaigns to examine the extent to which women's participation in tourism and sports reflects substantive transformations in opportunities and decision-making as opposed to primarily representational change.

In this way, the paper investigates whether Saudi women's increased participation represents a sustainable transformation in gendered power relations or remains largely performative. It seeks to answer three interrelated questions:

1. How are the image and role of Saudi women being reshaped in international tourism narratives in association with the 2034 World Cup?
2. To what extent are institutional mechanisms facilitating genuine empowerment vs. performative inclusion?
3. How do these initiatives function within the broader framework of Saudi cultural diplomacy under Vision 2030?

Through a critical socio-political lens grounded in theories of representation, the public sphere, and gender performativity, the study contributes to debates on gender, tourism, and mega-events by unpacking the symbolic and structural shifts at play in one of the most significant experiments in state-led transformation in the contemporary Middle East.

The role of Saudi women in tourism has attracted increasing scholarly attention in recent years, particularly as Vision 2030 positions female empowerment as a central pillar of national development. Workforce data indicate steady quantitative growth, with more than 66,000 women employed in tourism-related establishments by the end of 2020 mostly in food and beverage or customer-facing roles (General Authority for Statistics, 2022). Yet women remain underrepresented in higher-level managerial, strategic, and ownership positions. This pattern aligns with World Travel and Tourism Council (2023) findings showing that numerical gains in women's participation globally have not eliminated structural barriers to leadership access.

Research has therefore interrogated whether women's increasing presence signals substantive empowerment or an instrumental incorporation into state-led diversification and image rebranding. Pratiwi and Muslikhati (2024) argue that Vision 2030 communicates a narrative of modernity through women's visibility in hospitality and tourism, even as many continue to occupy symbolic or operational roles rather than positions of strategic influence. Their analysis highlights a lingering gap between representational inclusion and structural transformation.

Saudi-specific scholarship sharpens this distinction. Through a phenomenological examination of women employed in the tourism industry, Sobaih and Abu Elnasr (2024) identify persistent socio-cultural, organizational, institutional, and personal constraints that limit upward career mobility. Similarly, Alotaibi et al. (2025) find that while digital governance platforms such as Absher and Yesser have expanded women's procedural agency in travel, licensing, and services, cultural expectations continue to mediate the extent to which such autonomy is realized in everyday practice. Collectively, these studies show that enhanced access and visibility do not automatically translate into shifts in power relations.

Complementing contemporary analyses, earlier ethnographic and social studies illuminate the deeply contextual nature of women's spatial participation in Saudi Arabia. Le Renard (2014) documents how young women negotiate modernity and tradition through everyday practices, while Hamdan (2005) underscores education as both an instrument and a site of gendered negotiation. Le Renard (2008) and van Geel (2016) demonstrate that women's participation in public space is continuously shaped through localized, socially embedded negotiations around norms of visibility and presence. Rather than passive recipients of reform, Saudi women actively reinterpret boundaries between public and private spheres, an insight relevant to their roles in new leisure and tourism venues.

Tourism has also emerged as a key site of cultural diplomacy, where state-backed mega-projects such as AlUla, the Red Sea development, and NEOM prominently feature female staff in branding campaigns. While these representations signal progress, scholars caution that visibility within highly curated zones does not necessarily reflect structural inclusion across the sector. Such spatial selectivity raises theoretical and practical questions about which women are seen and where.

A major gap remains at the intersection of gender, tourism, and mega-events. Although research has examined women's employment trajectories and growing opportunities in local sports, little scholarship has addressed how global mega-events, particularly the 2034 FIFA World Cup, might shape symbolic and functional gender roles within tourism branding and national soft-power strategies. This gap is addressed by the current study, which extends the literature by analyzing how emergent sport-tourism futures may reconfigure female visibility, leadership, and cultural representation.

This study proposes a novel conceptual framework the mega-event gendered soft power model to examine how Saudi women's evolving participation in tourism and sports functions within the broader sociopolitical transformation shaped by Vision 2030 and the awarded hosting of the 2034 FIFA World Cup. While grounded in established theoretical traditions, the model advances a Saudi-specific lens that captures the interplay between representation, spatial agency, and institutional change.

Stuart Hall's (1997a,b) cultural representation theory provides the foundation for analyzing how mega-event discourses actively construct the figure of the “Saudi woman” as a signifier of national modernity. Rather than mirroring social change, promotional media selectively produce new femininities intended for both domestic consumption and global visibility. To assess how these constructions materialize in social space, the model draws on feminist revisions of the public sphere (Fraser, 1990). Mega-events create temporary spatial openings where gendered norms are renegotiated, enabling women to claim forms of presence historically restricted in Saudi Arabia whether as spectators in stadiums, guides in heritage sites, or hospitality professionals in globalized venues.

Crucially, spatial empowerment in the Saudi context does not merely entail physical access to the public domain. Rather, it functions as a process of spatial re-signification, whereby the symbolic meaning of place is actively renegotiated. As women occupy venues historically coded as male, such as sports arenas, tourism gateways, and heritage landscapes, the cultural scripts that define who belongs in those spaces begin to shift. These shifts generate a cumulative effect: institutions start to recognize and adopt these newly legitimized forms of presence in their regulatory and organizational practices. Through this dialectical interaction between space and institution, women's visibility can transition from representational inclusion to recognized institutional authority, reflected in expanded access to leadership and decision-making roles.

To capture this dynamic interplay, the mega-event gendered soft power model introduces three analytical mechanisms:

1. Symbolic Inclusion: Women are positioned as visible markers of openness and modernity in state-led branding.
2. Spatial Empowerment (as Spatial Re-signification): Public spaces become arenas in which new gendered identities are negotiated and normalized.
3. Institutional Conversion: This is the degree to which symbolic and spatial shifts culminate in structural empowerment and policy-level recognition.

Although the model is presented analytically through three dimensions, it does not assume a linear or uncontested progression from symbolic inclusion to structural empowerment. Instead, it conceptualizes mega-event gender representation as an iterative process shaped by feedback loops between institutional narratives, spatial practice, and social interpretation. Representations promoted through official tourism and sports discourse may be reinterpreted, negotiated, or unevenly accepted across regions and social settings. Symbolic inclusion can generate enabling effects while also producing localized hesitation, adjustment, or resistance that influences how institutional change develops over time. The model, therefore, treats discourse, space, and institutional response as mutually shaping forces rather than fixed stages along a one-way path.

**Figure 1:**
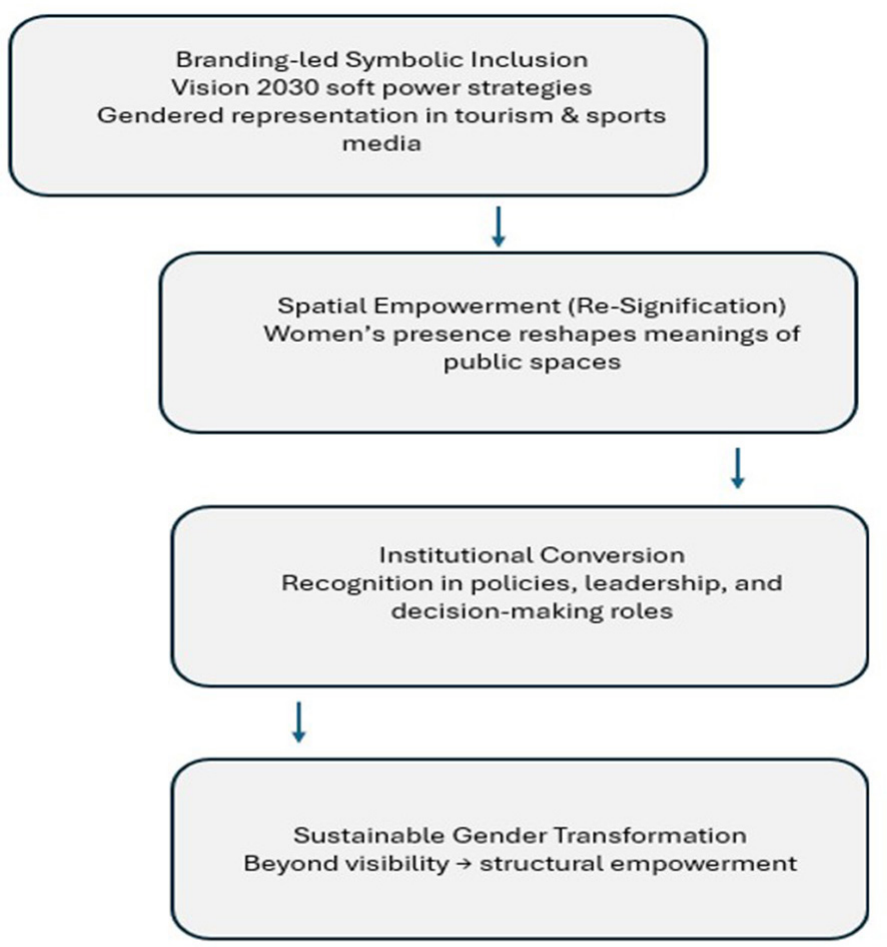
Mega-event gendered soft power pathway: from symbolic inclusion to structural empowerment.

Integrating these perspectives, the model offers an original theoretical contribution by situating Saudi women at the nexus of cultural representation, spatial transformation, and institutional restructuring. It provides a structured approach to assess whether global event-driven visibility fosters deeper empowerment or primarily serves soft-power objectives without redistributing authority. Accordingly, this framework positions gender not as a peripheral outcome of mega-event strategies but as a central arena where national identity and social change are actively co-produced. Figure 1 provides a visual representation of the framework.

## 2 Methodology

This study employs a qualitative, interpretive methodology grounded in critical discourse analysis (CDA) to examine how Saudi women are discursively positioned within mega-event tourism narratives associated with Vision 2030 and the 2034 FIFA World Cup. CDA provides an established analytical framework for understanding how visual and textual signs construct meaning and reproduce socio-cultural power relations within media and policy discourses (Fairclough, 1992). This approach is complemented by feminist cultural analysis informed by Hall's (1997a,b) cultural representation theory, Butler's (1990) work on gender performativity, and Fraser's (1990) revisions to the concept of the public sphere, enabling a deeper interrogation of how visibility, space, and legitimacy are constructed around women's participation in tourism and sports.

The analytical corpus consisted of a clearly delimited sample of 25 state-aligned media artifacts produced between 2022 and 2025, a period corresponding to the intensified international promotion of Saudi tourism and early communication phases related to the 2034 FIFA World Cup. The dataset included nationally circulated television advertisements, official promotional videos, and digital tourism communication materials published through institutional platforms such as Visit Saudi, the Ministry of Tourism, NEOM, and related official campaign outlets. The corpus was intentionally bounded to institutional media discourse to examine representational production rather than audience reception or everyday social practice. Accordingly, findings should be interpreted within the limits of official communication narratives rather than as a comprehensive account of societal change.

The empirical corpus consisted of 25 state-aligned media artifacts disseminated between 2022 and 2025 through official communication channels such as Visit Saudi, the Ministry of Tourism, NEOM, and FIFA-related promotional outlets. These included high-visibility broadcast advertisements, most notably, “This Land Is Calling” (Vision 2030, 2020–2024) and “Go Beyond What You Think” featuring Lionel Messi (Saudi Tourism Authority, 2024a,b), along with tourism branding videos, brochures, and digital promotion materials showcasing women in hospitality, heritage interpretation, and sports engagement roles. The selection of these materials was guided by their strategic deployment in national image-building and by their recurring focus on Saudi women as public representatives in emerging tourism spaces. These artifacts collectively constitute a discursive dataset that reflects state-sanctioned narratives about women's evolving roles in global participation. The study incorporates indirect data triangulation by interpreting discursive findings alongside existing empirical research and institutional data, allowing for cross-validation between representational patterns and independently observed tourism behaviors.

The predominance of government documents, official tourism campaigns, and state-affiliated media materials within the corpus reflects a deliberate analytical focus rather than a limitation of data availability. To mitigate potential concerns regarding the predominance of state-aligned materials, the dataset incorporated multiple forms of institutional discourse rather than relying solely on promotional campaigns or press releases. In addition to tourism advertisements and national branding videos, the broader evidentiary base included official sectoral reports, workforce statistics releases, institutional program descriptions, and policy-oriented communications produced by tourism and sport governance bodies. These materials comprised documents such as ministerial reports, tourism workforce statistics published by the General Authority for Statistics, program descriptions linked to training and certification initiatives in the tourism sector, and governance-related communications associated with Vision 2030 and major international event planning.

Including institutional texts from different categories allows the analysis to capture not only symbolic nation-branding narratives but also organizational and policy-level discourse through which gender participation is articulated, measured, or operationalized. This broader institutional scope strengthens the empirical base of the study while remaining consistent with its analytical focus on the production of official discourse, rather than audience reception or everyday social practice. The study examines how gender representation is discursively produced within nation-branding and mega-event communication, domains in which institutional actors play a primary role in shaping and circulating symbolic narratives. Accordingly, the dataset concentrated on officially disseminated materials where representations of Saudi women were strategically constructed for both domestic and international audiences. The analysis, therefore, addresses the production of discourse rather than its reception or the lived experiences of women in everyday contexts. While this institutional focus necessarily narrows the empirical scope, it allows for systematic examination of the level at which national image-making and soft-power messaging are intentionally formulated. Broader social interpretations and audience responses fall beyond the scope of the present study and are identified as important directions for future research.

Materials were selected using three predefined criteria aligned with the study's research objectives. To ensure analytical consistency, the criterion of “national visibility” was operationalized through a clear selection rule. Media artifacts were considered to have national visibility when they were officially published through a recognized national institutional platform (e.g., Visit Saudi, the Ministry of Tourism, NEOM, or other government-affiliated communication channels) and disseminated as part of a publicly accessible tourism or sport promotion initiative.

For analytical clarity, the dataset distinguishes between two levels of visibility: “baseline visibility” refers to materials that were published through an official institutional platform and circulated within national tourism communication channels; “high visibility” refers to materials that were additionally embedded within nationally coordinated campaigns or widely circulated branding initiatives associated with Vision 2030 tourism promotion or international event marketing.

This operational definition ensures that all selected artifacts meet a consistent threshold of institutional dissemination while allowing the analysis to differentiate between routine official communication and strategically amplified nation-branding campaigns. All materials included in the corpus satisfied the baseline visibility requirement, while a subset of artifacts classified as high-visibility campaigns received closer interpretive attention due to their strategic role in national tourism branding.

First, a high-visibility material had to demonstrate national visibility within Vision 2030 tourism or sport-related promotional narratives. Second, it had to include Saudi women appearing as active participants in public, professional, or representational roles within tourism, sports, hospitality, or cultural mediation contexts. Third, the content had to be published or officially disseminated by governmental or state-affiliated communication platforms between 2022 and 2025. These criteria ensured analytical consistency by focusing on media texts intentionally designed to project national identity and gender representation to domestic and international audiences. Materials were excluded when they did not contain visible representation of Saudi women, originated from unofficial or user-generated sources, or repeated identical visual sequences without introducing new representational elements. Social media commentary, journalistic opinion pieces, and audience responses were intentionally excluded to maintain analytical focus on institutional discourse production rather than reception. This exclusion strategy reduced sampling bias and preserved coherence between the research questions and the discursive nature of the dataset.

The study's analytical procedures followed Fairclough's (1992) three-dimensional model. First, a micro-textual and micro-visual examination identified semiotic cues including posture; gesture; attire; spatial orientation; camera angles; verbal scripts; and lexical markers signaling empowerment, authority, and cultural legitimacy. Second, discursive practices were analyzed by tracing circulation patterns, intended audiences, and integration with national branding strategies. Third, these representations were interpreted in relation to broader socio-political contexts, specifically, the transformation agenda embedded in Vision 2030.

All visual materials were segmented into micro-events, annotated frame-by-frame, and coded inductively using a structured scheme aligned with the theoretical framework. Codes such as *female agency, cultural continuity, spatial presence, hybrid identity*, and *soft-power positioning* emerged as dominant across the dataset. These first-level codes were then consolidated into higher-order categories corresponding to the following conceptual model: symbolic inclusion → spatial empowerment → institutional conversion.

In this study, institutional conversion is operationalized as an emergent inferential mechanism rather than a completed structural outcome. Given the study's primary reliance on publicly available media artifacts and future-oriented mega-event narratives, institutional conversion is assessed through discursive-institutional alignment, i.e., the extent to which women's symbolic and spatial visibility is accompanied by formal organizational recognition and policy-linked role articulation in authoritative institutional outputs. Empirically, this is examined using secondary institutional materials cited in the manuscript (e.g., ministerial reports, official statistics, program descriptions, and public institutional announcements) as institutional signals that corroborate whether representational inclusion is being translated into credentialing, role formalization, supervisory responsibility, or governance commitments.

Specifically, the analysis codes and interprets four observable indicators:

1. Role formalization (explicit institutional descriptions of women in supervisory/managerial or specialized roles);
2. Credentialing and pipeline formation (training, certification, and professional pathways tied to tourism/mega-event labor);
3. Policy and governance embedding (gender inclusion referenced as a criterion or commitment in event/tourism governance documentation); and
4. Monitoring and accountability language (references to measurable targets, indicators, or institutional evaluation systems).

These indicators allow the study to substantiate claims of institutional conversion within the limits of publicly accessible evidence, while distinguishing between symbolic visibility and institutionalized authority.

To make the assessment of institutional conversion traceable and open to evaluation, the four indicators are applied across two analytically distinct layers of material. Layer 1 consists of the core media corpus (*N* = 25 state-aligned tourism/sport promotional artifacts, 2022–2025). In this layer, the unit of coding is the document-level artifact (i.e., each advertisement/video/brochure counted as one unit), while visual segments were used only as internal evidence to justify the document-level coding decision. Layer 2 consists of secondary authoritative outputs (ministerial reports, official statistics releases, program announcements, and governance-related communications) that are already cited in the manuscript to contextualize tourism and sport-sector change. In this layer, the unit of coding is the document-level institutional output, with coding anchored in explicitly stated role descriptions, program requirements, governance language, or measurable targets when present. For each document in both layers, the four indicators—(1) role formalization, (2) credentialing and pipeline formation, (3) policy and governance embedding, and (4) monitoring and accountability language—are coded as “Present,” “Absent,” or “Indeterminate.” “Indeterminate” is used when women's participation is referenced in general terms without sufficient specificity to classify the indicator as present (e.g., mentions of participation without role-level detail). This procedure prevents interpretive inflation and distinguishes symbolic visibility from institutional recognition on the basis of observable textual evidence.

Table 1 presents a representative example of the coding structure, using the campaign “This Land Is Calling,” to illustrate how the analysis progresses from a surface-level description to an interpretive insight. For example, the low-angle framing of a woman in a modern abaya may be interpreted as signaling a hybridized form of authority rooted in cultural belonging. At the same time, the narration, “She welcomes the world to Arabia,” positions women as active mediators of cosmopolitan hospitality. Such semiotic choices underscore the narrative positioning of women not as passive symbols but as legitimate hosts of national identity within global tourism encounters. The matrix also reflects how references to heritage landscapes visually embed empowerment claims within continuity rather than rupture.

**Table 1:** CDA coding matrix for “This Land Is Calling” promotional video as an example.

Micro-segment (Frame-level event)	Visual/verbal cue	First-cycle codes	Analytical interpretation (Second-cycle coding insight)
Opening shot (low-angle framing of woman in modern abaya)	Authority-enhancing camera angle; Hybrid attire blending modern cut with abaya	Female agency; Hybrid identity; Visual authority	The framing constructs a culturally rooted yet modern representation of Saudi femininity, reinforcing symbolic legitimacy in public presence (Hall, 1997a,b).
Narration: “She welcomes the world to Arabia”	Female voice-over enacting hospitality and global openness	Nation-branding; Soft-power positioning; Cosmopolitan hospitality	Language and deixis assign women the role of mediators of national identity toward global audiences, signaling Vision 2030 soft-power ambitions.
Scenes of women near heritage monuments (e.g., petroglyphs, AlUla formations)	Cultural landmarks embedded as backdrop to women's movement through space	Heritage continuity; Cultural guardianship; Spatial belonging	Women are visually anchored within historical sites, producing discourses of authenticity and legitimizing spatial expansion in culturally sensitive domains.
Slow walking barefoot across desert	Unhurried motion; grounded bodily engagement with place	Embodied heritage; Spatial empowerment; Cultural resilience	The barefoot movement performs a rooted femininity aligned with national cultural continuity while symbolically expanding gendered spatial mobility.
Group interaction in public spaces (urban and touristic)	Normalized visibility; Mixed-context presence	Public sphere access; Everyday empowerment; Normalization of presence	Increased female presence signals redefinition of public sphere boundaries and gradual erosion of gender-segregated spatial norms (Fraser, 1990).
Closing scene with Vision 2030 branding	Policy-discursive cue linking transformation to state agenda	State-aligned modernization; Institutional legitimation	Representation becomes explicitly embedded within state transformation discourse, merging empowerment symbolism with governmental policy narratives.

Codes were developed inductively through first-cycle descriptive and *in-vivo* coding, then refined via theoretical axial coding grounded in CDA and feminist media theory (Butler, 1990; Fairclough, 1992).

To maintain analytic reflexivity and prevent imposed researcher bias, interpretive memoing was integrated into the process. These memos documented evolving insights into spatial performativity, symbolic gesture, and the transference of agency, such as the moment in “Go Beyond What You Think” where a teenage girl, rather than Messi breaks the barrier marked “Girls can't,” reconfiguring symbolic constraint into a projected visibility of athletic futures. This memoing practice strengthened conceptual alignment between emergent codes and the study's research questions on symbolic vs. structural empowerment.

Validity was strengthened through a form of interpretive triangulation across materials, theoretical grounding, and continuous cross-comparison of symbolic patterns. The interpretive design is particularly suited to analyzing future-oriented national branding strategies in which visual and textual representations precede lived transformations. Given that the 2034 World Cup remains forthcoming, CDA enables the examination of empowerment narratives as anticipatory constructions that seek to shape social expectations in advance. Accordingly, this methodology provides a rigorous framework for examining how women's visibility appears to be strategically constructed as part of a national project of soft-power projection.

Institutional conversion is operationalized through a set of discursive indicators that signal shifts from symbolic representation toward forms of institutional recognition. These include: (1) the presence of women in officially sanctioned public roles, (2) the normalization of female participation across diverse social spaces, and (3) the integration of gender-inclusive narratives within state-led development frameworks. While these indicators do not constitute direct evidence of structural transformation, they provide a basis for identifying early-stage signals of institutional reconfiguration within the limits of discourse analysis.

As a Saudi female researcher, my analytical perspective is shaped by both cultural proximity and scholarly distance from the social context examined in this study. This positionality provides contextual familiarity with symbolic practices, gender norms, and visual cues embedded in national media narratives, while also requiring deliberate reflexive awareness to avoid assuming shared meanings or normalizing interpretations. Throughout the analysis, reflexivity was maintained through continuous analytic memo writing, documentation of coding decisions, and the use of an audit trail linking visual segments to interpretive claims. Interpretations are therefore grounded in observable discursive features within the corpus rather than personal experience or normative assumptions. The study does not claim to represent the lived experiences of Saudi women; rather, it analyzes how institutional media construct representations of women within specific nation-branding and mega-event communication contexts.

This study followed established CDA protocols to ensure analytical rigor, transparency, and traceability. The selection of media artifacts was based on three explicit criteria: (1) national visibility within the Vision 2030 tourism narrative; (2) direct portrayal of Saudi women in public or professional contexts; and (3) publication by official entities such as the Saudi Tourism Authority, NEOM, and related government platforms between 2022 and 2025. The final sample consisted of 25 media items, including televised advertisements and digital promotional videos, which were retrieved from publicly accessible government channels. Table 2 presents a map of evidence for institutional convergence.

**Table 2:** Institutional conversion evidence map.

Indicator	Evidence source	Description	Coding
Role formalization	Ministry of Tourism, Saudi Arabia (2024a)	Women employed in tourism workforce roles	Limited
Credentialing	Bunyan Academy—Red Sea Global	Hospitality certification programs	Present
Governance embedding	FIFA 2034 bidding documentation	Gender inclusion commitments	Partial
Monitoring language	Saudi Press Agency (2024)	45% women workforce statistic	Monitoring

The analytical procedures were conducted in four structured phases:

1. Textual and Visual Segmentation: Each video was divided into micro-segments capturing key gestures, attire, spatial relations, and verbal cues. Still frames were extracted where needed to enhance visual precision.
2. Iterative Coding: First-cycle codes were assigned inductively (e.g., “female agency,” “hybrid identity,” or “spatial negotiation”), followed by axial coding that consolidated themes into higher-order categories grounded in the study's theoretical lenses (Butler, 1990; Fraser, 1990; Hall, 1997a,b).
3. Analytic Memo Writing: Reflective memos were produced immediately following each coding session, capturing emergent interpretive insights and refining thematic connections.
4. Theoretical Integration: Codes and memos were systematically mapped to Fairclough's (1992) three-dimensional CDA model, textual features, discursive practice, and sociocultural context, ensuring alignment with the research questions and interpretive framework.

Throughout the process, coding decisions were documented in a dynamic audit trail to ensure transparency and traceability. No artificial intelligence-assisted or automated analytic tools were used in the coding or interpretation stages; all coding and memo writing were conducted manually by the author.

Given the study's reliance on publicly available materials without personal or sensitive data, ethics approval was not required, in accordance with Frontiers' research integrity policy. All sources are cited within the manuscript, and links to primary media materials are provided in the reference list where publicly accessible. While the dataset is intentionally focused on state-aligned institutional discourse, this necessarily introduces limitations related to representativeness and potential bias, as it does not capture the full diversity of social experiences, regional variations, or audience interpretations. This limitation is acknowledged as an inherent feature of discourse-oriented research that prioritizes the analysis of representational production over reception or lived experience.

Table 3 presents a representative sample of the analyzed materials and illustrates how coding categories were applied across the dataset.

**Table 3:** Sample of analyzed materials and coding illustration.

ID	Material	Type	Key theme	Coding example
M1	“This Land Is Calling” (Visit Saudi, 2024)	Tourism campaign video	Cultural authenticity and national identity	Women positioned as cultural hosts
M2	“Go Beyond What You Think” (Saudi Tourism Authority, 2024)	Promotional video	Breaking stereotypes/global image	Female agency and symbolic empowerment
M3	AlUla Tourism Representation (Royal Commission for AlUla, 2023)	Cultural promotion	Heritage tourism and global engagement	Women as heritage interpreters

## 3 Results

### 3.1 Symbolic re-semantization of Saudi femininity

Across the analyzed media materials, Saudi women are strategically positioned as agents of cultural continuity rather than symbols of rupture. This representational pattern recurs in multiple materials, particularly those that combine heritage imagery, female visibility, and public-facing professional roles. The most recurrent visual logic, in which women wear modernized abayas while assuming public-facing professional roles, functions according to what (Hall 1997a,b) describes as the re-articulation of cultural signifiers. Instead of replacing heritage with modernity, the imagery re-semantizes tradition to accommodate women's evolving public presence.

This is particularly evident in campaigns such as “This Land Is Calling,” where the physical setting of desert dunes, ancient rock formations, and cultural icons anchors women's visibility within the symbolic core of Saudi identity. Such portrayals deploy cultural authenticity as a legitimizing resource, constructing female empowerment not as an imported discourse but as a continuity of national heritage. Through Butler's (1990) lens, these representations perform a new femininity that remains culturally grounded while enabling expanded spatial mobility, thereby increasing public acceptance.

Within this representational strategy, the Saudi woman is not exhibited as a deviation from societal norms; she is framed as one of their legitimate narrators. Accordingly, the transformation observed is not mere numerical inclusion and involves discursive reconstruction of who is entitled to perform national identity in public.

This symbolic re-semantization is consistent with recent national reporting by the Ministry of Tourism, which highlights that 45% of tourism-sector employees are now women, a statistic repeatedly framed as a marker of cultural continuity rather than Westernization (Saudi Press Agency, 2024). Similarly, the (U.S.-Saudi Business Council 2023) documented that the rise of women's workforce participation beyond Vision 2030 targets is narratively tied to “national values” and identity rather than external pressure. These institutional data points reinforce that women's visual and symbolic presence is state-endorsed and socially legitimized, not disruptive.

Aligned with (Hall 1997a,b), heritage-anchored symbolism becomes a crucial representational resource: women guide tourists at AlUla archaeological sites, appear at cultural festivals, and host international delegations—practices that embed female agency inside the national narrative rather than at its margins. Thus, empowerment is discursively framed as authentic and indigenous—a form of cultural authorship rather than cultural borrowing.

### 3.2 Spatial empowerment and controlled expansion of presence

The second emergent pattern concerns women's expanding access to the public sphere, particularly within tourism, sports, and high-visibility mega-event spaces. Here, Fraser's (1990) notion of pluralized public spheres is helpful: rather than a single liberated space, women's visibility grows in institutionally engineered zones where empowerment aligns with state-led development logics.

Mega-projects like NEOM and AlUla serve as pilot environments of spatial empowerment, where women appear confidently driving, guiding visitors, or in leading hospitality roles. These locales operate as cultural thresholds, that is, socially sanctioned spaces where femininity can be publicly enacted without challenging the broader social contract. The spatial negotiation is intentional, not incidental. On the one hand, women are allowed to expand where national ambitions and global gaze converge, particularly in contexts linked to the 2034 FIFA World Cup and tourism diplomacy. On the other, this pattern is not uniformly distributed across all materials. While high-visibility campaigns emphasize women's presence in globally oriented spaces, lower-visibility institutional materials tend to present more limited or role-specific forms of participation, indicating variation within the dataset.

Thus, spatial empowerment is real but uneven: expanding in global-facing innovation-oriented spaces, but restricted to locally conservative geographies. This pattern highlights a Saudi-specific theoretical contribution to empowerment advances first through state-curated spaces of visibility before diffusing socially. The result is a phased transformation, where inclusion is not driven by confrontation with tradition but by spatially anchored re-design of what counts as acceptable public presence.

Mega-projects and international sports partnerships further consolidate these spatial openings. For example, the (Royal Commission for AlUla 2023) publicly reported that women now hold visible roles in hospitality, tour guiding, and cultural interpretation linked to global tourism exhibitions in Paris, Venice, and Berlin. In sports, FIFA's 2023 confirmation of the World Cup awarding process explicitly references gender inclusion commitments as part of Saudi Arabia's hosting credentials (Fédération Internationale de Football Association, 2023).

Such initiatives create what Fraser (1990) would define as sub-public spheres, strategically positioned, globally visible zones where women enact spatial agency under state-constructed legitimacy. These curated terrains embody a transformation path unique to Saudi Arabia: empowerment flows from the center outward, beginning where international gaze and policy ambitions converge.

### 3.3 Symbolic capital and entrepreneurial agency

As Saudi Arabia expands its tourism ecosystem through giga-projects such as the Red Sea development, NEOM, and AlUla, a growing number of Saudi women are emerging not only as employees but also as entrepreneurial actors shaping the industry's direction. In particular, official sector reporting indicates a growing expansion of women's participation across licensed tourism activities and entrepreneurial initiatives within the Kingdom's tourism ecosystem (Ministry of Tourism, Saudi Arabia, 2024b; Saudi Press Agency, 2024). This shift reflects what Bourdieu (1986) conceptualizes as symbolic capital, the accumulation of status and legitimacy that transforms visibility into recognized authority.

In multiple policy case briefs, women-led tourism ventures are showcased as exemplars of localized innovation aligned with national priorities. For instance, the Bunyan Academy program developed in collaboration with Red Sea Global has trained Saudi women for high-profile hospitality roles, including the country's first certified female butlers (EHL Hospitality Business School, 2024). These profiles are more than isolated success stories and demonstrate how international credentials together with national rootedness produce a form of agency that is culturally validated and strategically amplified. Across the dataset (*N* = 25), the majority of analyzed materials consistently depict Saudi women in culturally grounded yet publicly visible roles, with recurring visual patterns including traditional attire combined with professional positioning, heritage settings, and direct engagement with international audiences.

Meanwhile, the Saudi Press Agency (2024) emphasizes that in tourism documents, women's entrepreneurship is not framed as a challenge to tradition but as a demonstration of women's contributions to economic prosperity “in alignment with national values.” This rhetorical framing strengthens public acceptance, enabling entrepreneurial women, such as licensed tour operators in heritage sites, to act as cultural ambassadors representing the nation to global visitors.

Consistent with Butler's (1990) performativity lens, these entrepreneurial roles introduce new performances of Saudi femininity as professional, authoritative, and globally oriented yet visibly anchored in national authenticity. Women's ventures in sectors like desert expeditions, culinary tourism, and cultural storytelling further reposition them as producers, not merely promoters, of the tourism experience.

While symbolic inclusion and spatial empowerment are most directly observable in the media corpus, institutional conversion is here assessed by tracing whether these representational shifts are accompanied by institutional signals of role formalization, credentialing, governance embedding, and monitoring language in authoritative outputs cited throughout the paper. Empirically, this assessment was conducted through the two-layer mapping procedure outlined above and summarized in Table 1. The table distinguishes between (a) media-level visibility claims that remain representational and (b) institutional texts that articulate role definitions, training/credentialing pathways, governance commitments, or measurable indicators. Where institutional outputs provided only aggregate participation figures without role differentiation, the evidence was coded as participation visibility rather than conversion. This distinction is analytically central: institutional conversion, as used here, requires that women's presence becomes legible to institutions as role-based authority, pipeline entitlement, governance criteria, or monitored targets, not merely as promotional imagery or headline participation metrics.

The analysis identifies a pattern of discursive-institutional alignment in which women's visibility as hosts, guides, and athletes is increasingly mirrored by institutional descriptions of women's participation across tourism and event-adjacent labor domains, including professional training pipelines and supervisory functions. These signals do not demonstrate a completed redistribution of power; rather, they indicate early-stage conversion whereby symbolic and spatial openings become legible within institutional categories of recognition and responsibility.

#### 3.3.1 Women's agency in iconic cultural and religious spaces

The expansion of women's roles in Hajj and Umrah administration represents one of the most consequential yet understudied dimensions of Saudi Arabia's socio-spatial transformation (General Authority for Statistics, 2025). Historically, pilgrimage governance was male-dominated due to religious custodianship and conservative norms. Yet Vision 2030 reforms have facilitated systematic inclusion of women in operational, managerial, and service domains across the sacred cities, in roles deeply intertwined with national identity and religious legitimacy. The gradual expansion of women's participation in operational and service roles associated with the most symbolically charged infrastructure in the Kingdom signals institutional confidence in women's competencies. It also marks a reconfiguration of spiritual labor previously coded as masculine.

From the perspective of Fraser's (1990) reconceptualization of the public sphere, the Haram is not merely a physical space but a moral-political arena. Thus, participation in its governance signifies access to one of the most authoritative domains of public life in the Islamic world. Women's emerging presence here represents a redistribution of spatial and symbolic power, extending public agency into religious sovereignty itself. Through Butler's (1990) gender performativity lens, this participation can be interpreted as the enactment of a legitimate religious femininity, where women's bodies and voices perform authority through service, guidance, and skill rather than desacralized visibility. The role is sacred, embodied, and nationally validated—a distinctly Saudi pathway to empowerment.

Simultaneously, these roles function as what (Hall 1997a,b) would classify as sites of representational renewal: the image of a Saudi woman directing international pilgrims, speaking multiple languages, or coordinating safety procedures becomes a discursive artifact that reshapes what “Saudi womanhood” signifies to global publics. Government initiatives have institutionalized this shift, transforming symbolic legitimacy into credentialed authority and mapping directly onto the proposed conceptual model's pathway: representation → spatial inclusion → institutionalization → structural authority.

Importantly, women's participation in pilgrimage operations bridges modern identity projection with religious continuity, reinforcing the state's message that empowerment is not a break from tradition but a culturally grounded evolution of it. In this way, Saudi women are demonstrating that sacred service can be both a national duty and a professional arena, positioning the religious tourism sector as a cornerstone of gendered transformation with global visibility.

### 3.4 Sports diplomacy and women's global visibility toward 2034

The intersection of women's participation in sports and Saudi Arabia's pursuit of international mega-events reveals a strategic use of gender visibility in soft-power diplomacy. Football in particular has become a symbolic stage through which Saudi Arabia articulates a modern national identity. Recent reporting citing industry research indicates that 61% of female sports fans in Saudi Arabia follow football (Arab News, 2025b). The Saudi Women's Premier League has grown in both team presence and public attention (Saudi Press Agency, 2024). These shifts suggest a noticeable reconfiguration of gendered participation within the Kingdom's most culturally influential sport.

Within the conceptual model developed in this study, such visibility represents a performativity of global belonging (Butler, 1990), where women embody a national narrative that aligns with global sporting norms while retaining culturally grounded aesthetics. State branding campaigns, including “Go Beyond What You Think” and “This Land Is Calling,” foreground women's physical confidence and public presence not as deviations from tradition but as evidence of a Saudi modernity authored from within. These narratives operationalize cultural representation (Hall, 1997a,b) in ways that convert symbolic inclusion into internationally legible soft-power capital.

As preparations intensify for the 2034 FIFA World Cup, women are increasingly positioned not merely as spectators but as front-facing cultural ambassadors: tourism hosts, hospitality managers, event organizers, and sports professionals whose roles enable the Kingdom to project a gender-inclusive image to visiting global publics. This institutional positioning reflects a spatial renegotiation of the public sphere (Fraser, 1990), where stadiums, fan zones, and tourism venues become newly accessible arenas for female mobility and authority.

However, this diplomatic potential remains contingent on transitioning from carefully curated visibility to structural empowerment. The challenge lies in ensuring that women are not confined to symbolic roles in marketing materials while decision-making domains, such as federations, event committees, and ownership structures, remain male-dominated. Sustainable empowerment requires institutional guarantees: leadership pathways, safe employment environments, and equitable policies embedded into the legal governance of mega-events. In effect, women's participation in football and World Cup-aligned tourism is emerging as a litmus test for the substantive depth of Vision 2030′s gender reforms, where the world will soon be watching the distinction between representation and real power.

Across the layers examined in this study—cultural representation, spatial participation, and sports diplomacy—a consistent pattern emerges: symbolic inclusion appears to be advancing faster than structural empowerment. This divergence aligns with findings from community-based tourism contexts, where gender inclusion becomes more likely to be transformative when participation is accompanied by economic engagement and sustained local integration (Nikjoo et al., 2025). Here, this pattern instead indicates a transitional phase in which representational expansion precedes the consolidation of institutional authority.

Drawing on Hall's (1997a,b) conception of cultural representation, the increasing visibility of Saudi women in media campaigns constructs a new semiotic architecture of national identity in which femininity is aligned with modernity, capability, and global belonging. Such symbolic positioning also expands what becomes institutionally actionable. When women occupy newly legitimized public spaces, as theorized through Fraser's (1990) rethinking of the public sphere, their presence challenges boundaries not through confrontation but through normalization. Stadiums, fan zones, airports, and UNESCO heritage sites become arenas of entitlement, where participation translates into sustained claims to voice, mobility, and authority. This shift is further reinforced through Butler's (1990) idea of performativity: every instance in which Saudi women appear as athletes, hosts, or cultural ambassadors constitutes a repeated act that gradually sediments into a new common sense of gender roles in Saudi society.

However, symbolic restructuring alone does not guarantee equitable power redistribution. The crucial question, especially as the 2034 FIFA World Cup approaches, is whether these discursive openings solidify into organizational pathways that grant women decision-making power, not merely representational presence.

Saudi Arabia's Vision 2030 framework already embeds structural commitments to equality in tourism, labor markets, and sports governance, providing a policy foundation upon which symbolic gains may translate into:

- Leadership pipelines in mega-event organizations;
- Sustainable employment across tourism value chains;
- Expanded regulatory protections and professional rights; and
- Spatial inclusivity beyond major urban centers.

Despite the expansion of visibility and participation, the analysis indicates variation in how these developments are distributed across contexts. In particular, opportunities for women's participation appear more prominently in high-visibility globally oriented tourism and event spaces, while other settings reflect more gradual forms of inclusion. This pattern suggests that the relationship between representation and institutional roles may differ across locations and sectors.

## 4 Discussion

This study set out to address a core research question: How are Saudi women being represented and positioned within tourism and sports narratives linked to Vision 2030 and the 2034 FIFA World Cup, and what do these narratives reveal about the broader trajectory of gender transformation in Saudi Arabia? Applying a theoretically informed CDA to national media campaigns, policy documents, and tourism strategies, the study suggests that women's increasing visibility in public space may function as a discursive mechanism contributing to the shaping of social expectations.

Through the lens of Hall's (1997a,b) theory of cultural representation, the findings reveal that portrayals of Saudi women often rely on symbolic markers such as traditional attire or heritage settings to preserve cultural continuity while signaling modern participation. Butler's (1990) theory of gender performativity helps interpret women's roles in sports and tourism as enactments of new femininities, where public visibility and embodied action contribute to redefining social norms. Meanwhile, Fraser's reconceptualization of the public sphere (1990) allows for an understanding of how tourism spaces, mega-events, and religious service roles are becoming contested arenas in which women claim voice, mobility, and recognition. Together, these theoretical perspectives illuminate how symbolic narratives function as preconditions for structural transformation, producing visibility that both responds to and accelerates policy reforms.

The study's original contribution lies in extending this theoretical dialogue to the understudied intersection of women, tourism, and mega-event diplomacy in Saudi Arabia. While existing research has examined women's empowerment in education, labor, or general social change, few studies have interrogated how tourism media and sports branding under Vision 2030 are reconfiguring gender identity and public spatial participation. Foregrounding women in global tourism narratives and positioning them as cultural ambassadors in the lead-up to the 2034 FIFA World Cup, Saudi Arabia is constructing a future-oriented vision of public womanhood that advances national branding while also negotiating internal cultural legitimacy. This multidimensional reframing, which is at once economic, symbolic, and spatial, marks a significant sociological shift with long-term implications. Empirical studies of tourism motivations further indicate that such transformations are reflected not only at the level of representation but also in gendered patterns of participation and preference (Alkohaiz et al., 2025).

However, symbolic advancements alone are not sufficient. The recommendations outlined below translate the theoretical insights into actionable strategies that align representational empowerment with institutional change, ensuring that media visibility materializes as sustainable participation. These recommendations support the balanced and culturally grounded modernization approach emphasized by Vision 2030 and reflect the principle of equality in empowerment adopted in national strategies.

The recommendations are derived directly from the empirical patterns identified in the discourse analysis rather than from general policy considerations. Across the corpus, women are most consistently positioned as visible cultural mediators and hosts of national authenticity (symbolic inclusion), while indicators of role formalization, leadership access, and accountability mechanisms appear less consistently and remain uneven across contexts (institutional conversion). In addition, the dataset shows geographically differentiated portrayals in which women's mobility and professional visibility are more strongly associated with metropolitan and showcase development zones than with peripheral regions (spatial unevenness). For these reasons, the recommendations focus on strengthening the conditions under which representational visibility is more likely to translate into institutional recognition through clearer professional pathways, measurable governance commitments, and regionally inclusive capacity-building while remaining aligned with the limits of what can be inferred from institutional media discourse.

### 4.1 Recommendations

#### 4.1.1 Expand capacity-building and training

Nationwide programs should be established to enhance women's skills in tourism, sports leadership, and cultural diplomacy, with tailored initiatives in heritage-rich and rural areas such as AlUla to ensure regionally balanced participation. The corpus repeatedly portrays women as hospitality hosts, cultural mediators, and tourism guides, particularly in globally visible development zones such as AlUla and the Red Sea. However, institutional discourse indicates that professional credentialing pathways remain unevenly distributed geographically. Strengthening structured training pipelines would help translate symbolic participation into regionally balanced professional capacity.

#### 4.1.2 Integrate gender-sensitive policies in tourism and mega-event governance

Work should be done to institutionalize inclusive policies such as transparent promotion pathways, leadership targets, and workplace protections to convert symbolic inclusion into durable equality. Media materials frequently position Saudi women as visible representatives of national hospitality, yet references to formal governance roles or decision-making authority are limited within the corpus. This discrepancy between representational visibility and institutional authority supports the need for governance frameworks that formalize women's participation in leadership and event management structures.

#### 4.1.3 Strengthen private sector commitments

Efforts should be made to encourage hospitality groups, tourism developers, and event managers to adopt gender inclusion standards through incentives such as recognition schemes, certification programs, and targeted public-private partnerships. Several institutional references highlight women's employment growth in tourism, while certain examples (e.g., hospitality training initiatives) demonstrate emerging partnerships between national institutions and international tourism actors. Expanding such collaborations would help convert individual success cases into sector-wide institutional practices.

#### 4.1.4 Develop aligned educational and vocational pathways

Initiatives should be adopted to enhance university and technical programs in the tourism, sports, and heritage sectors to create a sustainable pipeline of qualified female professionals aligned with future labor demands. The analysis identifies growing female participation in tourism-facing roles but fewer representations of women occupying specialized or strategic positions. This pattern suggests the importance of educational pipelines that support career mobility beyond operational roles.

#### 4.1.5 Improve cultural representation standards in media narratives

Diverse and authentic portrayals of Saudi women should be promoted, reinforcing both domestic legitimacy and international credibility, rather than relying primarily on symbolic archetypes. Across the corpus, women are frequently represented through culturally grounded imagery such as heritage landscapes, traditional attire, and hospitality symbolism. While these representations support cultural legitimacy, expanding the range of portrayed roles would reflect the broader spectrum of women's participation emerging in the tourism and sports sectors.

#### 4.1.6 Increase women's leadership roles across tourism ecosystems

Efforts should be made to boost representation in entrepreneurship, sports governance, and mega-event organizational structures. While the corpus highlights women as cultural ambassadors and service professionals, explicit portrayals of women in strategic leadership positions remain comparatively limited. Strengthening pathways into governance and entrepreneurial roles would align institutional authority with the growing visibility of women in public-facing sectors.

#### 4.1.7 Implement measurable monitoring and evaluation systems

Transparent indicators should be introduced to track participation across roles and regions, enabling evidence-based policy adjustments. Institutional discourse referenced in this study includes participation statistics and sector growth indicators; however, systematic monitoring frameworks linking visibility, employment, and leadership representation remain inconsistently articulated. Establishing measurable indicators would support the transition from symbolic inclusion to sustained institutional accountability.

### 4.2 Limitations and future research

While this study provides critical insights, it is limited by its reliance on publicly available media materials and secondary sources, without incorporating the lived experiences of women from diverse geographical and social contexts. Urban-centric portrayals may overshadow slower yet meaningful change in rural communities. Additionally, the analysis is temporally bounded by developments up to mid-2025, excluding more recent shifts in cultural or policy implementation.

Future research should incorporate mixed methods, including interviews, ethnographic fieldwork, and longitudinal studies, to examine how symbolic visibility translates into material empowerment over time. Comparative investigations between regions within Saudi Arabia, or between Saudi Arabia and other Gulf states, would provide a richer understanding of contextual variations. Moreover, emerging dynamics such as the influence of digital media, influencer culture, and grassroots sports movements deserve deeper attention as Saudi Arabia prepares for the global spotlight of the 2034 FIFA World Cup.

### 4.3 Theoretical contribution

This study proposes a new conceptual contribution: the mega-event gendered soft power model. The framework explains how women's visibility in tourism and sports media becomes a crucial intermediary stage between symbolic empowerment and structural transformation. It builds on Hall's (1997a,b) representation theory, Fraser's (1990) feminist public sphere, and Butler's (1990) gender performativity, while extending them to the specific context of Gulf mega-event modernization.

The framework identifies three interlocking dimensions: (1) women appear as cultural ambassadors rooted in heritage yet positioned within global identity narratives—and these representational shifts adjust social imaginaries and legitimize women's presence in formerly restricted spaces; (2) mega-event tourism produces new gendered public spaces; and (3) women's participation becomes geographically uneven accelerated in globalized urban areas and Vision 2030 showcase zones (e.g., Riyadh, Jeddah, and AlUla), while remaining slower in traditional regions.

For empowerment to become substantive, visibility must also convert into:

- Policy inclusion;
- Leadership authority;
- Career mobility; and
- Sectoral decision-making power.

In this way, symbolic capital functions as a precondition for structural empowerment, not a substitute for it. This framework is the first to map how global sports and tourism events reshape gendered subjectivities in Saudi Arabia, particularly in anticipation of the 2034 FIFA World Cup. It thus advances international debates on gender, nation-branding, and the sociology of mega-events by grounding them in a culturally specific, non-Western transformation model.

## Data Availability

The original contributions presented in the study are included in the article/supplementary material, further inquiries can be directed to the corresponding author.
